# Complete mitochondrial genome of stripped fruit fly, *Bactrocera (Zeugodacus) scutellata* (Diptera: Tephritidae) from Anshun, Southwest China

**DOI:** 10.1080/23802359.2017.1347899

**Published:** 2017-07-07

**Authors:** Jian-Hong Liu, Ping-Fan Jia, Lan-Lan Liu, Qing-Mei Wang, Yu-Xin Zhang, Shi-Qin Ruan

**Affiliations:** a Yunnan Provincial Key Laboratory of Forest Disaster Warning and Control, Southwest Forestry University, Kunming, China;; b School of Agriculture, Anshun University, Anshun, China

**Keywords:** *Bactrocera (Zeugodacus) scutellata*, mitochondrial genome, phylogeny

## Abstract

The striped fruit fly, *Bactrocera (Zeugodacus) scutellata* occurred in East and Southeast Asia and is one of the most serious pest insects in China. Complete mitochondrial genome was sequenced and characterized for the fruit fly from Anshun, Guizhou Province in Southwest China. The pest fruit fly has a total length of 15,904 bp, consisting of 13 protein-coding genes (PCGs), 22 tRNA, 2 rRNA genes and a non-coding region (A + T-rich control region). The phylogenetic position of *B. scutellata* was closely clustered with *B. cucurbitae, B. tau, B. caudata* and *B. diaphora*. The all species of the subgenus *Zeugodacus* formed a sister group which was monophyletic. The complete mitochondrial genome of *B. scutellata* will provide helpful information for genetics, systematics and phylogeny of tephritid fruit flies, Particularly *Bactrocera* genus.

The striped fruit fly, *Bactrocera scutellata*, is distributed in east and southeast Asia (Jeon et al. 2011). The species is recognized from other *Zeugodacus* fruit fly with three postsutural yellow vittae by possession of a pair of medium-sized circular black spots across the face, 2 pairs of scutellar bristles, and the costal band slightly enlarged at the apex (Sugimoto et al. 1988). The flowers and fruits of Cucurbitaceae were recorded as hosts for Larvae of *B. scutellata* (Ohno et al. 2006). In this study, we characterized the complete mitochondrial genome sequence of *B. scutellata* which could be used for studies on bio-identification, multi-mitochondrial gene-based phylogeny and biogeography.

Samples were collected from Anshun University campus located in Anshun, China (26°14′40″N, 105°54′7.09″E), in December 2016. Specimen was deposited in Yunnan Provincial Key Laboratory of Forest Disaster Warning and Control, Kunming, China. Genomic DNA was extracted following the manufacturer’s instruction in the DNeasy^®^ Blood and Tissue kit (Qiagen) for animal tissue (spin column). Using the total DNA as a template, two long PCR products were amplified, and then the shotgun library was constructed and the entire mitochondrial genome sequence was successfully obtained. Primers for the long fragments were designed from existing *Bactrocera* mitogenome sequences (Zhang et al. 2014; Choudhary et al. 2015; Yong et al. 2015, 2016; Jeong et al. 2017).

The pest fruit fly has a total length of 15,904 bp, consisting of 13 protein-coding genes (NAD1-6, NAD4l, COX1-3, CYTB, ATP6 and ATP8), 22 tRNA, 2 rRNA genes and a non-coding region (A + T-rich control region) (GenBank accession number: MF358969). The mitogenome length is well within the range in the completely sequenced genus *Bactrocera* mitochondrial genome available within the size from 15,815 bp in *B. oleae* (Nardi et al. 2003) to 16 043 bp in *B. minax* (Zhang et al. 2014). The nucleotide composition of *B. scutellata* mitochondrial genome is biased toward adenine and thymine (accounting for 73.0%: A = 39.3%, T = 33.7%, G = 10.0% and C = 17.0%). This bias is well within the range detected for the sequenced Tephritidae species, from 67.28% in *B. minax* to 77.48% in *Ceratitis capitate*.

The phylogenetic tree indicated that *B. scutellata* was closely clustered with *B. cucurbitae, B. tau, B. caudata* and *B. diaphora* (Figure 1). Among the component species of the subgenus *Zeugodacus* of the genus *Bactrocera* of tephritid fruit flies, distinct genetic lineages have been found based on complete mitochondrial genome sequences. The present finding of distinct genetic lineages in *B. scutellata* increases the number in the list. The phylogeny also showed that *B. carambolae* is closely related but distinct from *B. dorsalis, B. papaya, B. philippinensis* and *B. invaden* (Figure 1), three of which are highly similar, morphologically and genetically, to the destructive pest insect, oriental fruit fly, *B. dorsalis*. Furthermore, a recent study indicated that *B. papaya, B. philippinensis* and *B. invadens* had been synonymized with *B. dorsalis*, while *B. carambolae* was a valid species and belonged to *B. dorsalis* complex (Schutze et al., 2015).

**Figure 1.**
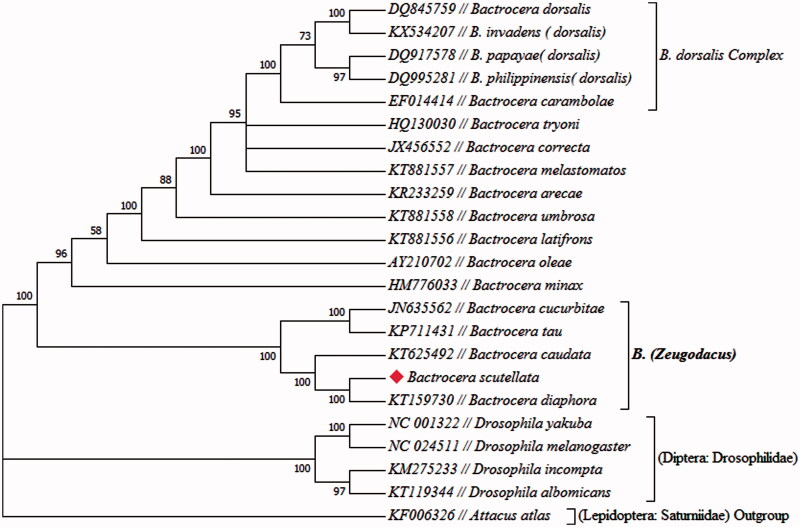
Maximum likelihood estimation of the phylogenetic relationship among speci-es. Genbank accessions were indicated with species name. *Bactrocera scutellata* was marked in solid rhombus shape.
